# Transcriptomic Changes in Young Japanese Males After Exposure to Acute Hypobaric Hypoxia

**DOI:** 10.3389/fgene.2020.559074

**Published:** 2020-09-08

**Authors:** Yoshiki Yasukochi, Sora Shin, Hitoshi Wakabayashi, Takafumi Maeda

**Affiliations:** ^1^Department of Human Functional Genomics, Advanced Science Research Promotion Center, Organization for the Promotion of Regional Innovation, Mie University, Tsu, Japan; ^2^Graduate School of Design, Kyushu University, Fukuoka, Japan; ^3^Faculty of Engineering, Hokkaido University, Hokkaido, Japan; ^4^Department of Human Science, Faculty of Design, Kyushu University, Fukuoka, Japan; ^5^Physiological Anthropology Research Center, Faculty of Design, Kyushu University, Fukuoka, Japan

**Keywords:** acclimatization, differentially expressed gene, hypobaric hypoxia, RNA-seq, saliva, transcriptome

## Abstract

After the genomic era, the development of high-throughput sequencing technologies has allowed us to advance our understanding of genetic variants responsible for adaptation to high altitude in humans. However, transcriptomic characteristics associated with phenotypic plasticity conferring tolerance to acute hypobaric hypoxic stress remain unclear. To elucidate the effects of hypobaric hypoxic stress on transcriptional variability, we aimed to describe transcriptomic profiles in response to acute hypobaric hypoxia in humans. In a hypobaric hypoxic chamber, young Japanese males were exposed to a barometric pressure of 493 mmHg (hypobaric hypoxia) for 75 min after resting for 30 min at the pressure of 760 mmHg (normobaric normoxia) at 28°C. Saliva samples of the subjects were collected before and after hypobaric hypoxia exposure, to be used for RNA sequencing. Differential gene expression analysis identified 30 significantly upregulated genes and some of these genes may be involved in biological processes influencing hematological or immunological responses to hypobaric hypoxic stress. We also confirmed the absence of any significant transcriptional fluctuations in the analysis of basal transcriptomic profiles under no-stimulus conditions, suggesting that the 30 genes were actually upregulated by hypobaric hypoxia exposure. In conclusion, our findings showed that the transcriptional profiles of Japanese individuals can be rapidly changed as a result of acute hypobaric hypoxia, and this change may influence the phenotypic plasticity of lowland individuals for acclimatization to a hypobaric hypoxic environment. Therefore, the results obtained in this study shed light on the transcriptional mechanisms underlying high-altitude acclimatization in humans.

## Introduction

High altitude is a hostile environment for humans owing to low oxygen availability through decreased barometric pressure (P_*B*_), and high-altitude illness such as acute mountain sickness in lowlanders can occur at altitudes above 2100 m (World Health Organization, 2012). While the conventional genome-wide association study is difficult to detect genes related to phenotypic plasticity in response to acute hypobaric hypoxic stress, the transcriptome analysis can provide a better understanding of the genetic mechanisms in response to the acute environmental stress. Therefore, the identification of the responsible genes using the transcriptome analysis is clinically important for the prevention of acute hypoxic insult because it could be helpful in identifying the therapeutic targets. The Andean, Ethiopian, and Tibetan highlanders have resided in the Andean Altiplano (South America), Semien Plateau (Africa), and Tibetan Plateau (East Asia), respectively. While all three populations have settled at high altitudes for millennia, these populations are considered to have adapted to high altitude environments via different genetic variants (Bigham, 2016). In 1990, ∼2.4 million Tibetans were estimated to reside at altitudes above 3500 m (Wu, 2001). In addition, it was estimated that ∼140 million people lived at altitudes above 2500 m across the world (Moore et al., 1998). Of these, 13.7, 1.7, and 20.7 million people were estimated to live in Ethiopia, Tibet, and South American Andes, respectively. Recent high-throughput genome sequencing studies have contributed to finding signals of positive natural selection acting on genes associated with hematological or pulmonary traits in people residing at high altitudes and revealed that genes in the hypoxia-inducible factor (HIF) pathway are responsible for high-altitude adaptation in highland populations (Bigham, 2016).

In particular, genetic polymorphisms of endothelial PAS domain protein 1 (*EPAS1*) and egl-9 family hypoxia inducible factor 1 (*EGLN1*), which play a pivotal role in the upstream of the HIF pathway, in Tibetan highlanders have been extensively investigated; for instance, the study subjects lived in the Qinghai province (∼4350 m) or the Tibet Autonomous Region (∼4300–4600 m) in China and the Solu-Khumbu region in Nepal (∼3440 m), or were the descendants living in Virginia and Utah in the United States (Simonson et al., 2010; Yi et al., 2010; Hanaoka et al., 2012; Huerta-Sánchez et al., 2014; Lorenzo et al., 2014; Xu et al., 2015). The previous studies have reported that *EPAS1* and *EGLN1* are responsible for a relatively low hemoglobin concentration, which may be diluted by increased plasma volume (Stembridge et al., 2019), in Tibetan highlanders because it protects against the development of high-altitude polycythemia due to elevated red blood cell volume, while positive natural selection may have acted independently on *EGLN1* in Tibetan and Andean highlanders (Bigham et al., 2010). Moreover, a candidate-gene approach study exhibited that genetic polymorphisms of *EGLN1* in Japanese lowlanders were associated with blood oxygen saturation variation during acute hypobaric hypoxia exposure, whereas putative adaptive alleles in highland populations may be susceptible to acute hypobaric hypoxia in the lowland individuals because of lower blood oxygen saturation levels (Yasukochi et al., 2018). The discrepancy of adaptability to hypobaric hypoxia is likely due to the differences between the genetic backgrounds of highland and lowland populations.

In addition to the effects of genetic polymorphisms on the physiological response to hypobaric hypoxia, transcriptional variation from a genome in an individual can provide phenotypic plasticity in response to the extreme environmental conditions, conferring acclimatization to rapid environmental changes. In recent years, transcriptomic profiles in response to hypoxic stress have been investigated in free-ranging or wild endothermic (Pan et al., 2017; Lim et al., 2019; Park et al., 2019; Qi et al., 2019) and model animals (Sharma et al., 2015; Xu et al., 2019). It is well known that hypoxic microenvironments profoundly affect cancer progression in humans (Jing et al., 2019). Therefore, transcriptome analyses of various human cell types (Ricciardi et al., 2008; Salman et al., 2017; Parodi et al., 2018; Klomp et al., 2020) including tumor cells (Abu-Jamous et al., 2017; Berglund et al., 2018; Lichawska-Cieslar et al., 2018) under hypoxic microenvironments have been conducted, yet there is little knowledge about the effects of acute hypobaric hypoxic stress on the transcriptional profiles of humans *in vivo*. This might be due to the limitation of experimental facility and it makes difficult to collect a fresh sample that is essential to obtain non-degraded RNA from a study subject *in vivo*, while we could address the issue by using a large-size hypobaric hypoxic chamber (∼67.3 m^3^). Here, to elucidate the effects of hypobaric hypoxic stress on transcriptional variability, we described the transcriptomic profiles of young Japanese males, who are not athlete and mountain-climber, in response to acute hypobaric hypoxic stress conditions. The results obtained from this study may help in understanding the molecular mechanisms of acute hypobaric hypoxic tolerance in modern humans, and the findings may be applied to treat acute hypoxic insults that occur at sea level.

## Materials and Methods

### Study Subjects

Three Japanese male students of Kyushu University (Fukuoka, Japan) participated in the present study (mean age ± standard deviation, 22 ± 1 years; height, 175.0 ± 9.3 cm; weight, 58.4 ± 6.9 kg; body mass index, 19.1 ± 2.7 kg/m^2^). The participants had no clinical problems and no medium- or long-term stay at high altitudes preceding the hypobaric hypoxic experiment. They had abstained from drinking alcohol, doing strenuous exercise, and smoking for 24 h before the experiment, and had been prohibited from drinking caffeine and from eating 2 h prior to entering the hypobaric hypoxic chamber (Research Center for Human Environmental Adaptation, Kyushu University, Fukuoka, Japan). The dimension of the column-shaped chamber is as follow: the diameter and depth are 3.5 m and 7 m, respectively (cylindrical volume, ∼67.3 m^3^).

The study protocol complied with the Declaration of Helsinki and was approved by the Clinical Research Ethics Review Committee of Mie University Hospital (Approval number U2019-016) and the Ethics Committee of the Faculty of Design, Kyushu University (Approval number 269).

### Hypobaric Hypoxic Experiment

The hypobaric hypoxic experiment was carried out with the subjects wearing undershorts and short-sleeve T-shirts (∼0.12 clo) at 28°C. The experiment was conducted in the following order of conditions: the P_*B*_ of 760 mmHg (equivalent to sea level) was maintained for 30 min; the P_*B*_ was gradually decreased to the P_*B*_ of 493 mmHg (equivalent to the altitude of 3500 m) for 30 min; the decreased P_*B*_ was maintained for 75 min, and then the P_*B*_ was recovered to the ambient atmospheric pressure at sea level. The air temperature was maintained at 28°C during the experiment and the total duration of the experiment was 135 min. The experimental protocol was designed on experience, with safety as the priority. Saliva samples of study subjects were collected using the Oragene^®^ RNA kit (DNA Genotek, Ottawa, Canada) before and after exposure to hypobaric hypoxia. To assess the basal transcriptional fluctuations under no-stimulus conditions, two saliva samples were continuously collected from the experimenter (YY), who is a non-smoker, under normobaric normoxia conditions on the same day, in the similar manner of sample collection for the hypobaric hypoxic experiment (abstinence from drinking alcohol and doing strenuous exercise for 1 day, and from eating and drinking caffeine 2 h before the experiment). To minimize the risk of RNA contamination, total RNA of these samples was extracted on the different days.

### RNA Sequencing and Data Processing

Total RNA was extracted from the subjects’ saliva samples with a volume ≥ 2 mL using either the RNeasy micro kit (Qiagen, Hilden, Germany) or the NucleoSpin^®^ RNA kit (TaKaRa Bio, Shiga, Japan) according to the manufacturers’ protocol. NucleoSpin^®^ RNA Clean-up XS (TaKaRa Bio) was used to purify and concentrate the diluted RNA if the amount of total RNA was insufficient for RNA sequencing (RNA-seq). The sequence library was prepared using the TruSeq^®^ Stranded Total RNA Library Prep kit (Illumina, San Diego, CA, United States) and was sequenced as 100-bp paired-end (PE) reads on an Illumina NovaSeq 6000 platform (Illumina). The library preparation and RNA-seq were performed by Macrogen (Seoul, South Korea).

We used the fastp ver. 0.19.7 (Chen et al., 2018) to remove the Illumina PE adapters, polyG tail (and polyX tail), and low quality reads with the following parameters: −q 15 −n 10 −t 1 −T 1 −l 20. The filtered PE reads were mapped to the human reference genome (GRCh38) with annotation information included in the gtf (gene transfer format) file from Ensembl GRCh38.96, using the STAR ver. 2.5.3 (Dobin et al., 2013) after the reference genome was indexed by the RSEM ver. 1.3.0 (Li and Dewey, 2011). The RSEM program was also used to estimate gene expression levels from the mapped BAM (binary alignment map) files.

### Differential Gene Expression Analysis

From the estimated expression abundance (read-count) data at the gene level, we filtered out genes with < 7 read counts across all samples for further analysis. We normalized the filtered count data and estimated the log2 fold changes (LFC) using the generalized linear models with a negative binomial distribution implemented in Bioconductor R packages DESeq2 ver. 1.22.2 (Love et al., 2014) or edgeR ver. 3.24.3 (Robinson et al., 2009; McCarthy et al., 2012). The significance of gene expression differences between the normobaric normoxia and hypobaric hypoxia conditions was examined by the Wald or exact tests, and the *p*-value was adjusted to the false discovery rate (FDR) using the Benjamin and Hochberg method (Benjamini and Hochberg, 1995) for multiple testing. We identified genes with an FDR of < 0.05 and a |LFC| of ≥ 1 as differentially expressed genes (DEGs). After the count read data were transformed to the log2 scale by the rlog function in DESeq2, we performed principal component (PCA) and Euclidean distance analysis to compare the transcriptome similarity among samples. The differential gene expression analysis described above was performed using R software ver. 3.5.3 (R Core Team, 2019) via RStudio ver. 1.2.5019 (RStudio Team, 2019).

### Gene-Set Enrichment and Pathway Analysis

We performed Gene Ontology (GO) enrichment analysis in the biological process category for the DEGs identified in the present study, using the Bioconductor R package topGO ver. 2.34.0 (Alexa et al., 2006; Alexa and Rahnenfuhrer, 2018) via the RStudio software and ClueGO ver. 2.5.4 (Bindea et al., 2009) and BiNGO ver. 3.0.3 (Maere et al., 2005) via Cytoscape ver. 3.7.1 (Shannon et al., 2003). The statistical significance of the enrichment was tested using Fisher’s exact and hypergeometric tests in the topGO and ClueGO/BiNGO, respectively (FDR < 0.05). After the gene symbol of DEGs was converted into the NCBI Entrez Gene ID using the Bioconductor R package biomaRt ver. 2.38.0 (Durinck et al., 2005, 2009), the converted IDs were mapped to the Kyoto Encyclopedia of Genes and Genomes (KEGG^1^) pathway. A functional network of GO terms was generated using the topGO R package. The functional interactions between the DEGs were predicted using GeneMANIA ver. 3.5.1 Cytoscape plugin (Montojo et al., 2010, 2014; Warde-Farley et al., 2010) via the Cytoscape software.

## Results

### Identification of Differentially Expressed Genes in the Hypobaric Hypoxic Experiments

**FIGURE 1:**
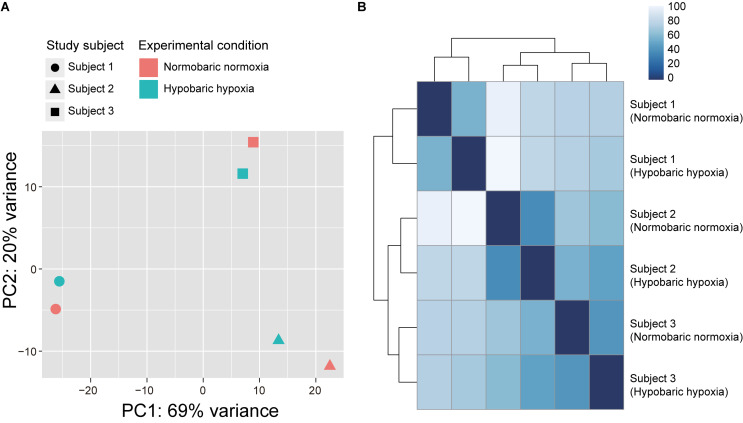
Transcriptome similarity among samples from three Japanese subjects participating in the acute hypobaric hypoxic experiments. **(A)** Principal component analysis of RNA-seq data plotted according to the first (horizontal axis) and second (vertical axis) principal components. **(B)** Heatmap of Euclidean distance between the expression vectors. The Euclidean distance was calculated using the R dist function.

A total of 307 million reads (51 ± 7 million reads) were obtained using RNA-seq from the six saliva samples belonging to the three Japanese individuals, to compare their transcriptomic profiles before and after exposure to acute hypobaric hypoxia. After quality control, 23 ± 3 million reads (18–26 million reads) were uniquely mapped to the human reference genome assembly. According to PCA (Figure 1A) and Euclidean distance analysis (Figure 1B) using the RNA-seq data, the expression vectors of the samples from the same individuals were clustered and formed three distinct groups of individuals, irrespective of the experimental conditions.

**FIGURE 2:**
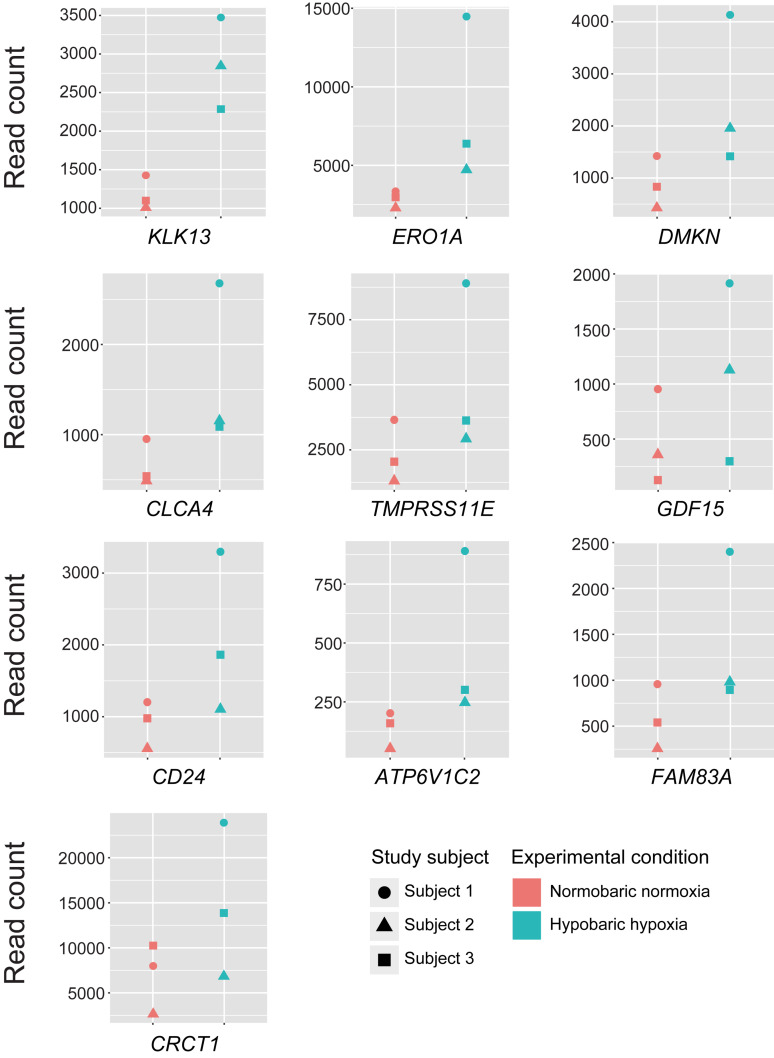
Expression levels of the top 10 differentially expressed genes in each of the study subjects. Read counts represent the estimated expression abundance at the gene level.

Using the DESeq2 program, we conducted the differential gene expression analysis and identified 30 DEGs being upregulated after exposure to acute hypobaric hypoxia (FDR < 0.05, LFC ≥ 1), while none of the significantly downregulated genes were detected (Table 1). The full gene name of these DEGs is listed in Supplementary Table S1. Of the upregulated 30 DEGs (mean LFC, 1.23 ± 0.18), 28 were protein-coding genes and the expression levels of at least 17 DEGs were possibly affected by hypoxic stress including tumor hypoxia or HIF-pathway activity in human cells or animal tissues (Supplementary Table S1). The remaining two DEGs (*LINC02487* and *AC005083.1*) were long noncoding RNA or processed transcript. We examined the differences in the expression levels among individuals in the top 10 ranked DEGs and found that the degree of transcriptional changes in each DEG varied within each individual (Figure 2).

**TABLE 1:** Thirty DEGs (FDR < 0.05, LFC ≥ 1) between normobaric-normoxic and hypobaric-hypoxic conditions in male Japanese subjects.

Gene symbol	LFC	Wald statistic	*p-*value	FDR	Gene biotype
*KLK13*	1.28	5.76	8.26 × 10^–9^	2.06 × 10^–5^	Protein coding
*ERO1A*	1.41	5.67	1.40 × 10^–8^	2.06 × 10^–5^	Protein coding
*DMKN*	1.49	5.33	9.65 × 10^–8^	9.45 × 10^–5^	Protein coding
*CLCA4*	1.24	5.17	2.40 × 10^–7^	1.66 × 10^–4^	Protein coding
*TMPRSS11E*	1.08	5.13	2.83 × 10^–7^	1.66 × 10^–4^	Protein coding
*GDF15*	1.29	4.85	1.22 × 10^–6^	5.97 × 10^–4^	Protein coding
*CD24*	1.12	4.76	1.96 × 10^–6^	8.24 × 10^–4^	Protein coding
*ATP6V1C2*	1.77	4.68	2.87 × 10^–6^	9.36 × 10^–4^	Protein coding
*FAM83A*	1.32	4.68	2.84 × 10^–6^	9.36 × 10^–4^	Protein coding
*CRCT1*	1.12	4.58	4.56 × 10^–6^	0.001	Protein coding
*KLK6*	1.55	4.54	5.75 × 10^–6^	0.001	Protein coding
*LINC02487*	1.43	4.46	8.28 × 10^–6^	0.002	LincRNA
*HEPHL1*	1.40	4.37	1.23 × 10^–5^	0.002	Protein coding
*S100A14*	1.01	4.36	1.27 × 10^–5^	0.002	Protein coding
*SBSN*	1.19	4.29	1.82 × 10^–5^	0.003	Protein coding
*CD177*	1.22	4.15	3.39 × 10^–5^	0.006	Protein coding
*PPP1R3C*	1.29	3.95	3.88 × 10^–5^	0.006	Protein coding
*SERPINB3*	1.29	3.89	8.28 × 10^–5^	0.012	Protein coding
*TMPRSS11A*	1.24	3.86	1.01 × 10^–4^	0.013	Protein coding
*ANKRD37*	1.21	3.85	1.19 × 10^–4^	0.013	Protein coding
*H1F0*	1.07	3.85	1.17 × 10^–4^	0.013	Protein coding
*PKP1*	1.09	3.82	1.33 × 10^–4^	0.014	Protein coding
*AQP3*	1.07	3.79	1.50 × 10^–4^	0.016	Protein coding
*LYPD3*	1.11	3.73	1.57 × 10^–4^	0.016	Protein coding
*CPA4*	1.04	3.70	2.18 × 10^–4^	0.019	Protein coding
*S100A7*	1.01	3.58	3.37 × 10^–4^	0.028	Protein coding
*GRPEL2*	1.25	3.53	4.22 × 10^–4^	0.033	Protein coding
*KLK12*	1.09	3.51	4.50 × 10^–4^	0.034	Protein coding
*AC005083.1*	1.27	3.44	5.92 × 10^–4^	0.039	Processed transcript
*ATG9B*	1.00	3.39	6.92 × 10^–4^	0.045	Protein coding

**DEG, differentially expressed gene; FDR, false discovery rate; LFC, Log2 fold change; LincRNA, long non-coding RNA.**

The differential gene expression analysis with the edgeR program detected 48 DEGs (36 upregulated and 12 downregulated genes) and 19 of the DEGs were also identified in the analysis with DESeq2 (Supplementary Table S2). However, |LFC| values of several DEGs were exceptionally high (> 7) whereas the read counts were low (Supplementary Figure S1). We thus used the 30 DEGs identified by DESeq2 for further analyses because the results obtained from this program were more conservative.

### Assessment of Baseline Variability in Gene Expression Levels During Normobaric Normoxia

To confirm basal transcriptomic profiles under no-stimulus conditions, we carried out the RNA-seq analysis using two saliva samples collected from the experimenter (YY) under normobaric normoxic environments on the same day, in the similar manner of the hypobaric hypoxic experiment. We obtained a total of 120 million reads (60 ± 4 million reads), and after quality control 27 million reads (26–28 million reads) were uniquely mapped to the human reference genome. To detect the DEG, the differential gene expression analysis was implemented in the edgeR program. The differential gene expression analysis indicated no significant difference (FDR > 0.05) in the gene expression levels during normobaric normoxic conditions, even though edgeR detected more DEGs than DESeq2 in the expression analysis for the hypobaric hypoxic experiment. Therefore, we used the 30 DEGs identified by DESeq2 for further analyses.

### Gene Function Enrichment Analysis

Using the 30 DEGs identified, we carried out GO enrichment analysis with topGO, BiNGO, and ClueGO programs, identifying a total of 145 significantly overrepresented GO terms (FDR < 0.05) based on the biological process category (Supplementary Tables S3–S5). Of these 145 GO terms, three related to the regulation of monocyte or granulocyte chemotaxis were consistently identified by all the enrichment analyses (Supplementary Figure S2). In addition, two and one GO terms were shared between BiNGO and topGO, and between ClueGO and topGO, respectively. These terms were also mainly related to the function of white blood cell subtypes. In our enrichment analyses, the GO terms associated with immunological or inflammatory processes accounted for a relatively high proportion of the enrichment analysis.

**FIGURE 3:**
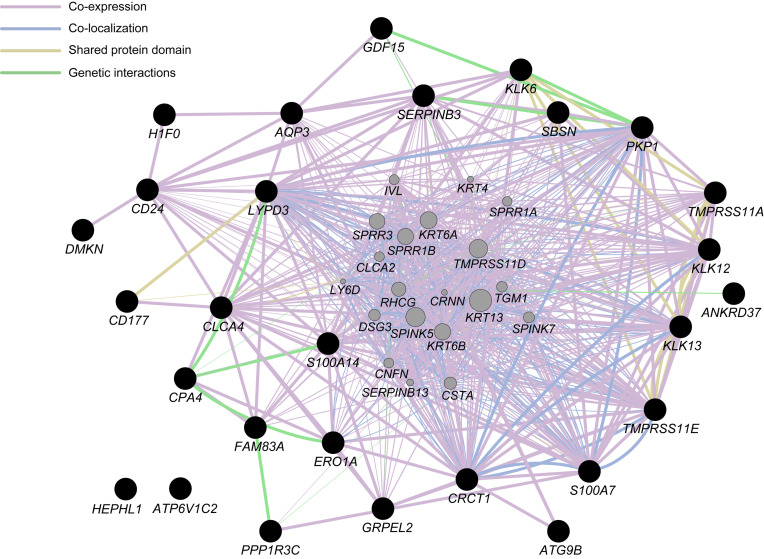
Network analysis for differentially expressed genes (DEGs), with the exception of long non-coding RNA or processed transcript, identified in the present study (closed black circles) with the use of GeneMANIA ver. 3.5.1 Cytoscape plugin via Cytoscape ver. 3.7.1. Interactions between the DEGs are indicated by bold lines. Genes represented by gray circles are putative mediators of the interactions between DEGs.

We also conducted the KEGG pathway analysis to predict the molecular function of the DEGs identified. The DEGs were mapped to 23 KEGG pathways although the number of DEGs included in each pathway was 1–2 only (Supplementary Table S6), and some of the KEGG pathways appeared to be related to functions in the immune system. Moreover, the hierarchical tree of GO terms overrepresented in the topGO analysis exhibited a significant relation of DEGs to migration and to the activation of several white blood cell types that are responsible for immune functions (Supplementary Figure S3). To further investigate the functional interactions among the DEGs, with the exception of long noncoding RNA or processed transcript, we performed a GeneMANIA network analysis (Figure 3). The network displayed potential direct or indirect interactions between the DEGs: many DEGs were coexpressed or colocalized and several pairs of DEGs shared a protein domain or directly interacted with each other.

## Discussion

The 30 DEGs were significantly upregulated in the three Japanese male subjects after exposure to acute hypobaric hypoxia. Under no-stimulus conditions, no significant transcriptional changes were observed, suggesting that the identified 30 DEGs in the Japanese individuals were actually upregulated owing to hypobaric hypoxic stress. Of these DEGs, several genes have been reported to be involved in response to hypoxic environments in previous studies using human cells and animal tissues.

The *ANKRD37* gene has been identified as a HIF target gene (Benita et al., 2009) and has a relatively early response of gene expression to hypoxic exposure (within 120 min) in human endothelial cells (Klomp et al., 2020), although the protein function encoded by *ANKRD37* remains to be elucidated. Our study demonstrated that the *ANKRD37* expression was significantly upregulated in the Japanese individuals after acute hypobaric hypoxia exposure for 75 min, and thus this gene may play an important role in response to hypobaric hypoxic stress at an early stage. We also found a significant upregulation of *S100A7* involved in various cellular processes such as proliferation and differentiation. Psoriasin, which is encoded by this gene, in keratinocytes was previously upregulated by hypoxic stress, and the expression patterns may affect several regulators of angiogenesis (Vegfors et al., 2016), suggesting that *S100A7* contributes to the hematological response to hypobaric hypoxia exposure in humans. In our study, *ATG9B* was also significantly upregulated in the Japanese individuals. It has been reported that the *ATG9B* transcript downregulates the nitric oxide synthase 3 (*NOS3*) transcript, and these gene expression levels in human endothelial cells can be changed by hypoxic environments (Kalinowski et al., 2016). Given that *NOS3* is responsible for the regulation of vascular wall homeostasis through production of nitric oxide with anti-inflammatory properties in the vascular endothelium (Fish and Marsden, 2006), *ATG9B* may be important for the regulation of hemodynamics in response to hypobaric hypoxic exposure. The expression levels of several other DEGs also appeared to be influenced by hypoxic stress in human cells (see Supplementary Table S1). In addition to the DEGs related to the hypoxic transcriptional changes in human cells, the gene expression of *HEPHL1* and *SBSN* was previously upregulated under hypoxic environments in zebrafish (Long et al., 2015) and sheep (Goyal and Longo, 2014), respectively. It has been reported that physiological responses such as ventilation were different between normobaric hypoxia and hypobaric hypoxia exposure (Coppel et al., 2015). This might be due to more severe oxidative stress under hypobaric hypoxia environments (Ribon et al., 2016); thus, the decrease of P_*B*_ may enhance the upregulation of genes involved in the production of reactive oxygen species (ROS) and antioxidant enzyme activities due to cellular oxidative damage. Indeed, the gene expression level of *ERO1A*, which is related to the ROS production (Nguyen et al., 2011), was significantly upregulated in the present study. Taken together, these results suggest that several DEGs identified in the present study are present in biological pathways relevant to physiological responses such as the alteration of hemodynamics and immune dynamics during hypoxic exposure, although the functional relevance of the DEGs to the physiological responses in humans remains unclear.

The GO enrichment and pathway analysis implemented in this study suggested that several DEGs were involved in immunological or inflammatory reactions during hypobaric hypoxia exposure. Given that the HIF pathway regulates the immune and inflammatory functions (Taylor and Colgan, 2017), the biological processes estimated from the DEGs may contribute to the altered immune cell activity through HIF-related pathways affected by exposure to acute hypobaric hypoxia. Although the KEGG pathway analysis indicated that few DEGs were involved in the same biological pathway, the GeneMANIA network analysis exhibited possible functional interactions among several DEGs. One may hypothesize that these DEGs participate in novel biological processes in response to hypobaric hypoxia exposure; alternatively, several biological processes including the DEGs might be activated simultaneously under hypobaric hypoxia.

However, transcriptional fluctuations observed in this study were limited within the same individual although we confirmed that the transcriptional patterns were altered by the hypobaric hypoxic experiments. This suggests that acute hypobaric hypoxic stress did not greatly affect the transcriptome profile of the study subjects, and it may result in no detection of significantly downregulated DEGs. It is possible that peaks of gene expression in response to hypoxia are varied and depend on the duration of hypoxic exposure (Klomp et al., 2020), while human DNA methylation levels, which can regulate gene expression, may be influenced by the severity or the duration of hypoxic exposure (Childebayeva et al., 2019). In addition, we focused on transcriptional changes after acute hypobaric hypoxia exposure but did not assess them during the exposure. This might decrease the statistical power to detect DEGs. Thus, further comprehensive analyses of RNA-seq data obtained from samples at different time points during the hypobaric hypoxic experiments are required. Also, it is necessary to collect the RNA sample for basal transcriptomic profiles under the no-stimulus condition from the hypobaric hypoxic experiment subjects.

In the present study, the magnitudes of transcriptional changes in each of the top 10 ranked DEGs under hypobaric hypoxia were relatively greater in the subject 1 than in the subjects 2 and 3. Given that the transcriptomic profile of the subject 1 appeared to be different from the other subjects in the PCA analysis, one may hypothesize that the transcriptional fluctuation of the subject 1 is different from the other subjects and may be susceptible to hypobaric hypoxic exposure. However, we cannot rule out the possibility to overestimate the transcriptional changes in the subject 1. It will be intriguing to examine interindividual variability of transcriptional changes in response to hypobaric hypoxic exposure, with a larger sample size.

The comparison of transcriptomic profiles before and after the alteration of environmental conditions is useful to evaluate phenotypic plasticity in response to environmental changes because the alteration of gene expression levels can be quantified. However, attention should be paid to the fact that gene expression levels are not necessarily correlated with protein levels, and that phenotypic plasticity can be affected by various factors including intrinsic characteristics of individuals and lifestyles. In addition, epigenetic modifications can affect gene expression levels. Further analyses for proteomic and epigenetic profiles are required to elucidate the molecular mechanisms underlying high-altitude acclimatization in humans.

In conclusion, we identified 30 DEGs being upregulated after acute hypobaric hypoxia exposure in Japanese lowlanders, and the transcriptional patterns of several genes can be altered owing to the exposure. This alteration may confer phenotypic plasticity in response to short-term hypoxic environmental changes; thus, it will be intriguing to examine differences in the transcriptomic profiles of groups of individuals showing similar physiological responses to acute hypobaric hypoxia. The results obtained in this study contribute to a better understanding of the physiological acclimatization of present-day lowlander humans to hypobaric hypoxia environments.

## Data Availability Statement

The datasets presented in this article are not readily available because of ethical and privacy issues. However, scientifically motivated requests for data sharing will be considered by reviewing the ethical committee of our institute.

## Ethics Statement

The studies involving human participants were reviewed and approved by the Clinical Research Ethics Review Committee of Mie University Hospital (Approval No. U2019-016) and the Ethics Committee of the Faculty of Design, Kyushu University (Approval No. 269). The participants provided their written informed consent to participate in this study.

## Author Contributions

TM conceived and designed the research. YY contributed to the research design, carried out the molecular lab work, participated in the analysis and interpretation of the data, and drafted the manuscript. TM and SS carried out the hypobaric hypoxic experiments and participated in the saliva sampling and data acquisition. TM, HW, and SS contributed to the interpretation of the data and the revision of the manuscript. All authors have read and approved the final manuscript.

## Conflict of Interest

The authors declare that the research was conducted in the absence of any commercial or financial relationships that could be construed as a potential conflict of interest.

## Footnotes

1 https://www.genome.jp/kegg/
