# Supplementary material for: Transcriptomic Changes in Young Japanese Males After Exposure to Acute Hypobaric Hypoxia
Source: Front Genet. 2020 Sep 8;11:559074. doi: 10.3389/fgene.2020.559074 (PMC7506118; doi:10.3389/fgene.2020.559074)
Supplement: Supplementary file 1 [file Presentation_1.pdf]

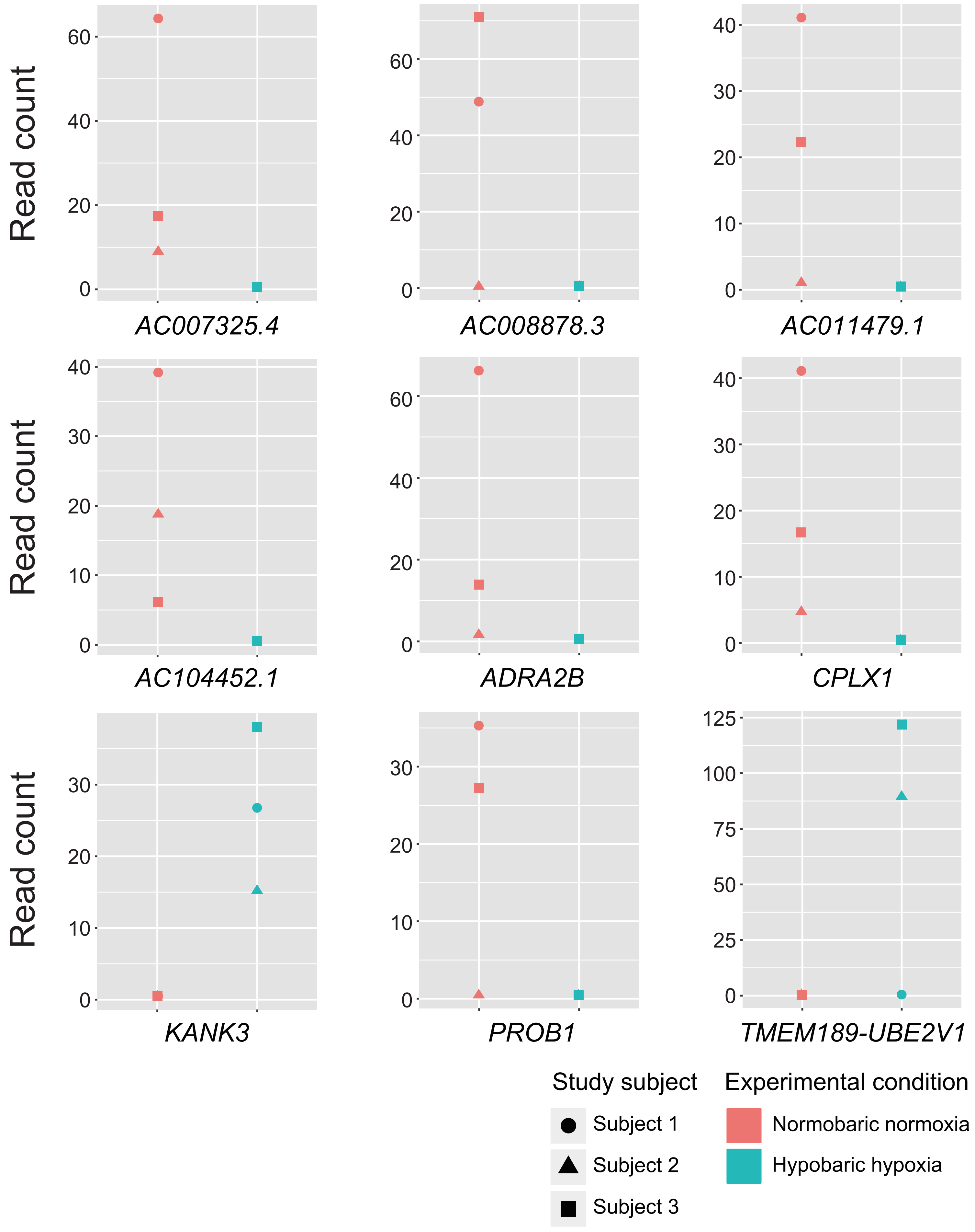

Supplementary Figure S1. Expression levels of the nine differentially expressed genes with |LFC| of > 7, identified by the edgeR program, in each of the study subjects. Read counts represent the estimated expression abundance at the gene level.

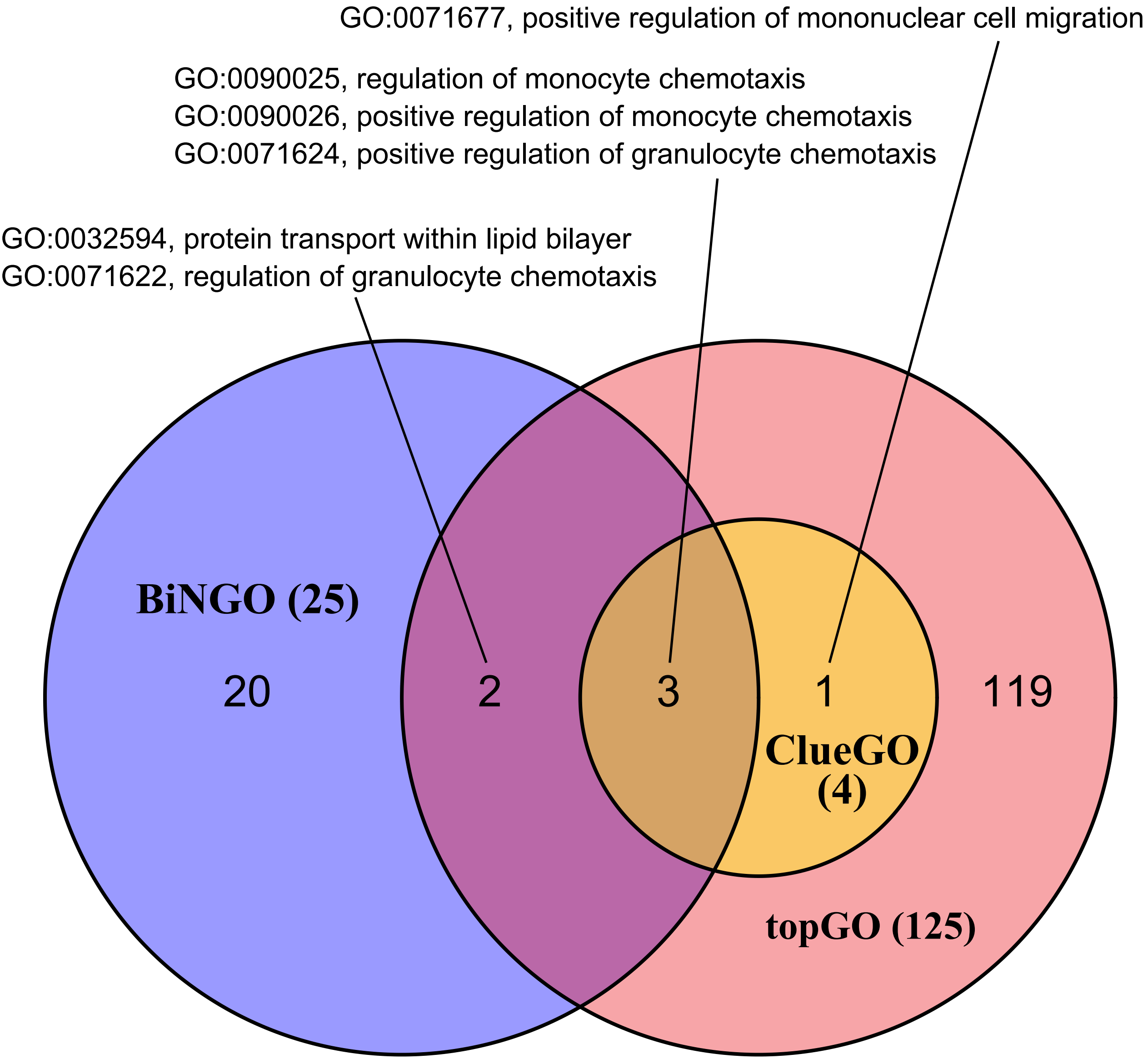

Supplementary Figure S2. Venn diagram of Gene Ontology terms estimated from 30 differentially expressed genes using three enrichment analysis methods.

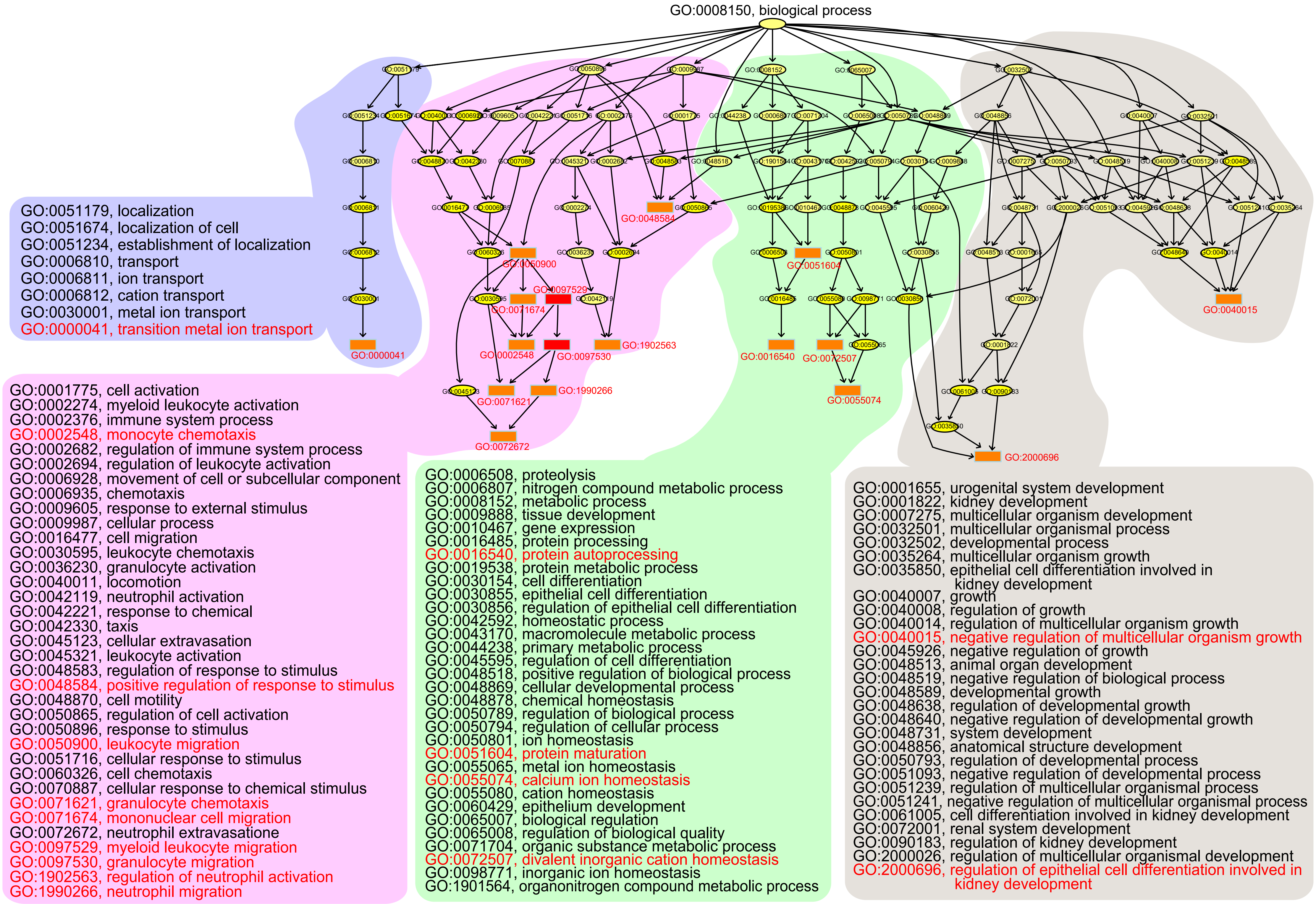

Supplementary Figure S3. Hierarchical tree of the overrepresented Gene Ontology terms (shown in red characters) estimated from 30 differentially expressed genes.
