# Supplementary material for: Transcriptomic Changes in Young Japanese Males After Exposure to Acute Hypobaric Hypoxia
Source: Front Genet. 2020 Sep 8;11:559074. doi: 10.3389/fgene.2020.559074 (PMC7506118; doi:10.3389/fgene.2020.559074)
Supplement: Supplementary file 2 [file Data_Sheet_1.pdf]

Supplementary Table S1. The 30 differentially expressed genes identified in this study.

Gene symbol	Full gene name	Reference[a]
AC005083.1	AC005083.1	
ANKRD37	ankyrin repeat domain 37	Benita et al., 2009; Klomp et al., 2020
AQP3	aquaporin 3 (Gill blood group)	Zieseniss et al., 2016
ATG9B	autophagy related 9B	Robb et al., 2004; Kalinowski et al., 2016
ATP6V1C2	ATPase H+ transporting V1 subunit C2	McConnell et al., 2017
CD24	CD24 molecule	Fujikuni et al., 2014
CD177	CD177 molecule	
CLCA4	chloride channel accessory 4	Ganaie et al., 2019
CPA4	carboxypeptidase A4	
CRCT1	cysteine rich C-terminal 1	
DMKN	dermokine	Huang et al., 2017
ERO1A	endoplasmic reticulum oxidoreductase 1 alpha	Takei et al., 2017
FAM83A	family with sequence similarity 83 member A	
GDF15	growth differentiation factor 15	Song et al., 2012; Haase, 2013; Gassmann and Muckenthaler, 2015
GRPEL2	GrpE like 2, mitochondrial	
H1F0	H1 histone family member 0	
HEPHL1	hephaestin like 1	Long et al., 2015
KLK6	kallikrein related peptidase 6	Yoon et al., 2007
KLK12	kallikrein related peptidase 12	Kryza et al., 2014
KLK13	kallikrein related peptidase 13	
LINC02487	long intergenic non-protein coding RNA 2487	
LYPD3	LY6/PLAUR domain containing 3	Ngora et al., 2012
PKP1	plakophilin 1	
PPP1R3C	protein phosphatase 1 regulatory subunit 3C	Shen et al., 2010
S100A7	S100 calcium binding protein A7	Vegfors et al., 2016
S100A14	S100 calcium binding protein A14	
SBSN	suprabasin	Goyal and Longo, 2014
SERPINB3	serpin family B member 3	Cannito et al., 2015
TMPRSS11A	transmembrane serine protease 11A	
TMPRSS11E	transmembrane serine protease 11E	

[a] The gene expression levels appeared to be affected by the hypoxic environment or by hypoxia-inducible factors in previous studies described below.

References

Benita, Y., Kikuchi, H., Smith, A. D., Zhang, M. Q., Chung, D. C., and Xavier, R. J. (2009). An integrative genomics approach identifies Hypoxia Inducible Factor-1 (HIF-1)-target genes that form the core response to hypoxia. *Nucleic Acids Res.* 37, 4587–4602. doi:10.1093/nar/gkp425.

Cannito, S., Turato, C., Paternostro, C., Biasiolo, A., Colombatto, S., Cambieri, I., et al. (2015). Hypoxia up-regulates SERPINB3 through HIF-2α in human liver cancer cells. *Oncotarget* 6, 2206–2221. doi:10.18632/oncotarget.2943.

Fujikuni, N., Yamamoto, H., Tanabe, K., Naito, Y., Sakamoto, N., Tanaka, Y., et al. (2014). Hypoxia-mediated CD24 expression is correlated with gastric cancer aggressiveness by promoting cell migration and invasion. *Cancer Sci.* 105, 1411–1420. doi:10.1111/cas.12522.

Ganaie, A. A., Parray, A., Hussain, T., Shabaneh, A., Jamroze, A., Ferrari, M. G., et al. (2019). Characterization of novel murine and human PDAC cell models: Identifying the role of intestine specific homeobox gene *ISX* in hypoxia and disease progression. *Transl. Oncol.* 12, 1056–1071. doi:10.1016/j.tranon.2019.05.002.

Gassmann, M., and Muckenthaler, M. U. (2015). Adaptation of iron requirement to hypoxic conditions at high altitude. *J. Appl. Physiol.* 119, 1432–1440. doi:10.1152/japplphysiol.00248.2015.

Goyal, R., and Longo, L. D. (2014). Acclimatization to long-term hypoxia: gene expression in ovine carotid arteries. *Physiol. Genomics* 46, 725–734. doi:10.1152/physiolgenomics.00073.2014.

Haase, V. H. (2013). Regulation of erythropoiesis by hypoxia-inducible factors. *Blood Rev.* 27, 41–53. doi:10.1016/j.blre.2012.12.003.

Huang, C., Xiang, Y., Chen, S., Yu, H., Wen, Z., Ye, T., et al. (2017). Dermokine contributes to epithelial–mesenchymal transition through increased activation of signal transducer and activator of transcription 3 in pancreatic cancer. *Cancer Sci.* 108, 2130–2141. doi:10.1111/cas.13347.

Kalinowski, L., Janaszak-Jasiecka, A., Siekierzycka, A., Bartoszewska, S., Woźniak, M., Lejnowski, D., et al. (2016). Posttranscriptional and transcriptional regulation of endothelial nitric-oxide synthase during hypoxia: the role of microRNAs. *Cell. Mol. Biol. Lett.* 21, 16. doi:10.1186/s11658-016-0017-x.

Klomp, J., Hyun, J., Klomp, J., Pajcini, K., Rehman, J., and Malik, A. B. (2020). Comprehensive transcriptomic profiling reveals SOX7 as an early regulator of angiogenesis in hypoxic human endothelial cells. *J. Biol. Chem.*, jbc.RA119.011822. doi:10.1074/jbc.RA119.011822.

Kryza, T., Achard, C., Parent, C., Marchand-Adam, S., Guillon-Munos, A., Iochmann, S., et al. (2014). Angiogenesis stimulated by human kallikrein-related peptidase 12 acting *via* a platelet-derived growth factor B-dependent paracrine pathway. *FASEB J.* 28, 740–751. doi:10.1096/fj.13-237503.

Long, Y., Yan, J., Song, G., Li, X., Li, X., Li, Q., et al. (2015). Transcriptional events co-regulated by hypoxia and cold stresses in Zebrafish larvae. *BMC Genomics* 16, 385. doi:10.1186/s12864-

015-1560-y.

McConnell, M., Feng, S., Chen, W., Zhu, G., Shen, D., Ponnazhagan, S., et al. (2017). Osteoclast proton pump regulator Atp6v1c1 enhances breast cancer growth by activating the mTORC1 pathway and bone metastasis by increasing V-ATPase activity. *Oncotarget* 8, 47675–47690. doi:10.18632/oncotarget.17544.

Ngora, H., Galli, U. M., Miyazaki, K., and Zöller, M. (2012). Membrane-bound and exosomal metastasis-associated C4.4A promotes migration by associating with the $\alpha_6\beta_4$ integrin and MT1-MMP[1,2]. *Neoplasia* 14, 95–107. doi:10.1593/neo.111450.

Robb, G. B., Carson, A. R., Tai, S. C., Fish, J. E., Singh, S., Yamada, T., et al. (2004). Post-transcriptional regulation of endothelial nitric-oxide synthase by an overlapping antisense mRNA transcript. *J. Biol. Chem.* 279, 37982–37996. doi:10.1074/jbc.M400271200.

Shen, G.-M., Zhang, F.-L., Liu, X.-L., and Zhang, J.-W. (2010). Hypoxia-inducible factor 1-mediated regulation of PPP1R3C promotes glycogen accumulation in human MCF-7 cells under hypoxia. *FEBS Lett.* 584, 4366–4372. doi:10.1016/j.febslet.2010.09.040.

Song, H., Yin, D., and Liu, Z. (2012). GDF-15 promotes angiogenesis through modulating p53/HIF-1α signaling pathway in hypoxic human umbilical vein endothelial cells. *Mol. Biol. Rep.* 39, 4017–4022. doi:10.1007/s11033-011-1182-7.

Takei, N., Yoneda, A., Sakai-Sawada, K., Kosaka, M., Minomi, K., and Tamura, Y. (2017). Hypoxia-inducible ERO1α promotes cancer progression through modulation of integrin-β1 modification and signalling in HCT116 colorectal cancer cells. *Sci. Rep.* 7, 1–11. doi:10.1038/s41598-017-09976-7.

Vegfors, J., Ekman, A.-K., Stoll, S. W., Bivik Eding, C., and Enerbäck, C. (2016). Psoriasin (S100A7) promotes stress-induced angiogenesis. *Br. J. Dermatol.* 175, 1263–1273. doi:10.1111/bjd.14718.

Yoon, H., Laxmikanthan, G., Lee, J., Blaber, S. I., Rodriguez, A., Kogot, J. M., et al. (2007). Activation profiles and regulatory cascades of the human kallikrein-related peptidases. *J. Biol. Chem.* 282, 31852–31864. doi:10.1074/jbc.M705190200.

Zieseniss, A., Hoogewijs, D., Vogler, M., Zwenger, E., and Krull, S. (2016). Oxygen-dependent regulation of aquaporin-3 expression. *Hypoxia* 4, 91–97. doi:10.2147/hp.s97681.

Supplementary Table S2. The 48 DEGs (FDR < 0.05, |LFC| ≥ 1) identified by the edgeR program.

Gene symbol	LFC	*p* value	FDR
TMEM189-UBE2V1	9.32	2.39×10^{-12}	2.87×10^{-8}
ERO1A[a]	1.60	3.78×10^{-8}	2.27×10^{-4}
CLCA4[a]	1.35	3.34×10^{-7}	0.001
KLK13[a]	1.31	3.38×10^{-7}	0.001
PLA2G4D	4.00	2.87×10^{-7}	0.001
ATP6V1C2[a]	1.85	5.09×10^{-7}	0.001
AC008878.3	-8.48	5.63×10^{-7}	0.001
DMKN[a]	1.50	1.85×10^{-6}	0.003
CD24[a]	1.22	3.24×10^{-6}	0.004
TMPRSS11E[a]	1.16	4.19×10^{-6}	0.005
SERPINA3	1.75	4.02×10^{-6}	0.005
TMPRSS11A[a]	1.45	4.72×10^{-6}	0.005
SERPINB3[a]	1.40	5.61×10^{-6}	0.005
GDF15[a]	1.26	6.47×10^{-6}	0.006
AC011462.1	1.83	9.60×10^{-6}	0.008
PPP1R3C[a]	1.44	1.17×10^{-5}	0.009
DENND2C	1.71	1.28×10^{-5}	0.009
FAM83A[a]	1.31	1.64×10^{-5}	0.011
AC005083.1[a]	1.59	1.97×10^{-5}	0.012
RASA4B	2.28	2.55×10^{-5}	0.015
AC007325.4	-8.04	2.68×10^{-5}	0.015
AQP3[a]	1.28	3.01×10^{-5}	0.016
HEPHL1[a]	1.35	3.03×10^{-5}	0.016
CPA4[a]	1.16	3.57×10^{-5}	0.018
ACP7	2.85	4.20×10^{-5}	0.019
KANK3	7.90	4.21×10^{-5}	0.019
IGHG1	-1.83	4.64×10^{-5}	0.021
ADRA2B	-7.88	5.59×10^{-5}	0.024
HSPH1	1.41	5.96×10^{-5}	0.025
MT-ATP8	1.22	6.55×10^{-5}	0.026
TMEM184A	1.42	7.72×10^{-5}	0.030
CYC1	-1.68	8.11×10^{-5}	0.030
KLK6[a]	1.53	9.62×10^{-5}	0.035
LINC02487[a]	1.30	1.01×10^{-4}	0.036
GABRE	1.33	1.31×10^{-4}	0.044
SPINK7	1.03	1.33×10^{-4}	0.044
CRCT1[a]	1.11	1.39×10^{-4}	0.044
HHIPL1	2.08	1.41×10^{-4}	0.044
CCL3	-1.09	1.45×10^{-4}	0.045
AC011479.1	-7.56	1.54×10^{-4}	0.045
FAM43A	1.42	1.60×10^{-4}	0.045
ALDH1A3	1.03	1.62×10^{-4}	0.045
PROB1	-7.53	1.62×10^{-4}	0.045

CCL2	-1.34	1.64×10^{-4}	0.045
APBB3	1.04	1.76×10^{-4}	0.047
IL6	-2.04	1.81×10^{-4}	0.047
AC104452.1	-7.50	1.85×10^{-4}	0.047
CPLX1	-7.49	1.93×10^{-4}	0.048

DEG, differentially expressed gene. FDR, false discovery rate. LFC, Log2 fold change.

[a] The DEG was also identified by the DESeq2 program.

Supplementary Table S3. GO processes estimated by topGO gene enrichment analysis using the 30 DEGs identified in this study.

GO ID	FDR	Description
GO:0097530	0.0004	granulocyte migration
GO:0097529	0.0013	myeloid leukocyte migration
GO:0002548	0.0020	monocyte chemotaxis
GO:0071674	0.0034	mononuclear cell migration
GO:0051604	0.0043	protein maturation
GO:0048584	0.0059	positive regulation of response to stimulus
GO:0000041	0.0063	transition metal ion transport
GO:1990266	0.0072	neutrophil migration
GO:0055074	0.0080	calcium ion homeostasis
GO:0071621	0.0080	granulocyte chemotaxis
GO:0072507	0.0103	divalent inorganic cation homeostasis
GO:0050900	0.0107	leukocyte migration
GO:1902563	0.0133	regulation of neutrophil activation
GO:0040015	0.0133	negative regulation of multicellular organism growth
GO:2000696	0.0133	regulation of epithelial cell differentiation involved in kidney development
GO:0016540	0.0133	protein autoprocessing
GO:0072672	0.0133	neutrophil extravasation
GO:0072160	0.0146	nephron tubule epithelial cell differentiation
GO:0061318	0.0146	renal filtration cell differentiation
GO:0072112	0.0146	glomerular visceral epithelial cell differentiation
GO:0072311	0.0146	glomerular epithelial cell differentiation
GO:0045217	0.0146	cell-cell junction maintenance
GO:2001044	0.0160	regulation of integrin-mediated signaling pathway
GO:0072010	0.0173	glomerular epithelium development
GO:0019471	0.0173	4-hydroxyproline metabolic process
GO:0002021	0.0173	response to dietary excess
GO:0036149	0.0173	phosphatidylinositol acyl-chain remodeling
GO:0034331	0.0173	cell junction maintenance
GO:0043410	0.0184	positive regulation of MAPK cascade
GO:1901741	0.0186	positive regulation of myoblast fusion
GO:0042755	0.0186	eating behavior
GO:0032930	0.0186	positive regulation of superoxide anion generation
GO:0036148	0.0186	phosphatidylglycerol acyl-chain remodeling
GO:0048245	0.0199	eosinophil chemotaxis
GO:0034975	0.0199	protein folding in endoplasmic reticulum
GO:0006575	0.0210	cellular modified amino acid metabolic process
GO:0090026	0.0212	positive regulation of monocyte chemotaxis

GO:1901739	0.0212	regulation of myoblast fusion
GO:0060396	0.0212	growth hormone receptor signaling pathway
GO:0071378	0.0212	cellular response to growth hormone stimulus
GO:0009967	0.0216	positive regulation of signal transduction
GO:0055065	0.0222	metal ion homeostasis
GO:0036150	0.0225	phosphatidylserine acyl-chain remodeling
GO:0008219	0.0230	cell death
GO:0072677	0.0239	eosinophil migration
GO:0060143	0.0239	positive regulation of syncytium formation by plasma membrane fusion
GO:0032928	0.0239	regulation of superoxide anion generation
GO:0006825	0.0239	copper ion transport
GO:0016485	0.0251	protein processing
GO:0071677	0.0252	positive regulation of mononuclear cell migration
GO:0043302	0.0252	positive regulation of leukocyte degranulation
GO:0030595	0.0254	leukocyte chemotaxis
GO:0060142	0.0265	regulation of syncytium formation by plasma membrane fusion
GO:0046475	0.0265	glycerophospholipid catabolic process
GO:0090025	0.0265	regulation of monocyte chemotaxis
GO:0042104	0.0265	positive regulation of activated T cell proliferation
GO:0071709	0.0278	membrane assembly
GO:0045745	0.0278	positive regulation of G protein-coupled receptor signaling pathway
GO:0071624	0.0291	positive regulation of granulocyte chemotaxis
GO:0036152	0.0291	phosphatidylethanolamine acyl-chain remodeling
GO:0055080	0.0293	cation homeostasis
GO:0010647	0.0297	positive regulation of cell communication
GO:0007204	0.0298	positive regulation of cytosolic calcium ion concentration
GO:0023056	0.0303	positive regulation of signaling
GO:0010831	0.0304	positive regulation of myotube differentiation
GO:0036151	0.0304	phosphatidylcholine acyl-chain remodeling
GO:0098771	0.0305	inorganic ion homeostasis
GO:0060416	0.0317	response to growth hormone
GO:0046471	0.0317	phosphatidylglycerol metabolic process
GO:0090184	0.0330	positive regulation of kidney development
GO:0042554	0.0330	superoxide anion generation
GO:0032594	0.0330	protein transport within lipid bilayer
GO:0007169	0.0331	transmembrane receptor protein tyrosine kinase signaling pathway
GO:0043406	0.0337	positive regulation of MAP kinase activity
GO:0007520	0.0343	myoblast fusion
GO:0002888	0.0343	positive regulation of myeloid leukocyte mediated immunity
GO:0044091	0.0343	membrane biogenesis
GO:0006658	0.0343	phosphatidylserine metabolic process

GO:0098660	0.0353	inorganic ion transmembrane transport
GO:0035850	0.0356	epithelial cell differentiation involved in kidney development
GO:0051480	0.0360	regulation of cytosolic calcium ion concentration
GO:0010862	0.0369	positive regulation of pathway-restricted SMAD protein phosphorylation
GO:0045730	0.0369	respiratory burst
GO:0050801	0.0373	ion homeostasis
GO:0030001	0.0381	metal ion transport
GO:0051085	0.0382	chaperone cofactor-dependent protein refolding
GO:0090322	0.0382	regulation of superoxide metabolic process
GO:0015991	0.0382	ATP hydrolysis coupled proton transport
GO:0071375	0.0387	cellular response to peptide hormone stimulus
GO:0071634	0.0395	regulation of transforming growth factor beta production
GO:0030574	0.0395	collagen catabolic process
GO:0034142	0.0408	toll-like receptor 4 signaling pathway
GO:0034220	0.0408	ion transmembrane transport
GO:0009395	0.0408	phospholipid catabolic process
GO:0043408	0.0409	regulation of MAPK cascade
GO:0060326	0.0411	cell chemotaxis
GO:0001666	0.0414	response to hypoxia
GO:0071604	0.0420	transforming growth factor beta production
GO:0050873	0.0420	brown fat cell differentiation
GO:0061005	0.0420	cell differentiation involved in kidney development
GO:0055085	0.0420	transmembrane transport
GO:0046006	0.0433	regulation of activated T cell proliferation
GO:0033572	0.0433	transferrin transport
GO:0015988	0.0433	energy coupled proton transmembrane transport, against electrochemical gradient
GO:0036293	0.0445	response to decreased oxygen levels
GO:0071675	0.0446	regulation of mononuclear cell migration
GO:0051084	0.0446	'de novo' posttranslational protein folding
GO:0090183	0.0446	regulation of kidney development
GO:0006654	0.0446	phosphatidic acid biosynthetic process
GO:0097193	0.0458	intrinsic apoptotic signaling pathway
GO:0000768	0.0459	syncytium formation by plasma membrane fusion
GO:0140253	0.0459	cell-cell fusion
GO:1903307	0.0459	positive regulation of regulated secretory pathway
GO:0015682	0.0459	ferric iron transport
GO:0072512	0.0459	trivalent inorganic cation transport
GO:0046473	0.0459	phosphatidic acid metabolic process
GO:0006949	0.0472	syncytium formation
GO:0071622	0.0472	regulation of granulocyte chemotaxis
GO:0060393	0.0485	regulation of pathway-restricted SMAD protein phosphorylation

GO:0061098	0.0485	positive regulation of protein tyrosine kinase activity
GO:0050798	0.0485	activated T cell proliferation
GO:0032835	0.0485	glomerulus development
GO:0002696	0.0494	positive regulation of leukocyte activation
GO:0043300	0.0497	regulation of leukocyte degranulation
GO:0006458	0.0497	'de novo' protein folding

GO terms with an FDR of < 0.05 and a minimum of 2 associated genes per GO term were defined as significantly overrepresented terms.

GO, gene ontology. DEG, differentially expressed gene. FDR, false discovery rate.

Supplementary Table S4. GO processes estimated by BiNGO gene enrichment analysis using the 30 DEGs identified in this study.

GO ID	*p* value	FDR	Description
GO:0071624	1.86×10^{-6}	7.78×10^{-4}	positive regulation of granulocyte chemotaxis
GO:0090025	1.11×10^{-5}	0.002	regulation of monocyte chemotaxis
GO:0090026	1.11×10^{-5}	0.002	positive regulation of monocyte chemotaxis
GO:0071622	2.78×10^{-5}	0.003	regulation of granulocyte chemotaxis
GO:0006508	3.59×10^{-4}	0.028	proteolysis
GO:0002684	4.41×10^{-4}	0.028	positive regulation of immune system process
GO:0002690	4.62×10^{-4}	0.028	positive regulation of leukocyte chemotaxis
GO:0002688	6.87×10^{-4}	0.029	regulation of leukocyte chemotaxis
GO:0002237	6.98×10^{-4}	0.029	response to molecule of bacterial origin
GO:0002687	8.43×10^{-4}	0.029	positive regulation of leukocyte migration
GO:0030856	9.55×10^{-4}	0.029	regulation of epithelial cell differentiation
GO:0032913	0.001	0.029	negative regulation of transforming growth factor-beta3 production
GO:0033606	0.001	0.029	chemokine receptor transport within lipid bilayer
GO:0032594	0.001	0.029	protein transport within lipid bilayer
GO:0032595	0.001	0.029	B cell receptor transport within lipid bilayer
GO:0032596	0.001	0.029	protein transport into membrane raft
GO:0032597	0.001	0.029	B cell receptor transport into membrane raft
GO:0032599	0.001	0.029	protein transport out of membrane raft
GO:0032600	0.001	0.029	chemokine receptor transport out of membrane raft
GO:0002685	0.001	0.029	regulation of leukocyte migration
GO:0050921	0.001	0.029	positive regulation of chemotaxis
GO:0048520	0.002	0.037	positive regulation of behavior
GO:0050920	0.002	0.041	regulation of chemotaxis
GO:0002682	0.003	0.044	regulation of immune system process
GO:0071635	0.003	0.047	negative regulation of transforming growth factor-beta production

GO, gene ontology. DEG, differentially expressed gene. FDR, false discovery rate.

Supplementary Table S5. GO processes estimated by ClueGO gene enrichment analysis using the 30 DEGs identified in this study.

GO ID	*p* value	FDR	Description
GO:0071677	8.29×10^{-4}	0.002	positive regulation of mononuclear cell migration
GO:0090025	0.001	0.001	regulation of monocyte chemotaxis
GO:0071624	0.001	0.002	positive regulation of granulocyte chemotaxis
GO:0090026	4.95×10^{-4}	0.002	positive regulation of monocyte chemotaxis

GO, gene ontology. DEG, differentially expressed gene. FDR, false discovery rate.

Supplementary Table S6. KEGG pathway estimated using the 30 DEGs identified in this study.

KEGG ID	Pathway	Gene name[a]
hsa00190	Oxidative phosphorylation	*ATP6V1C2*
hsa01100	Metabolic pathways	*ATP6V1C2*
hsa04060	Cytokine-cytokine receptor interaction	*GDF15*
hsa04136	Autophagy - other	*ATG9B*
hsa04137	Mitophagy - animal	*ATG9B*
hsa04140	Autophagy - animal	*ATG9B*
hsa04141	Protein processing in endoplasmic reticulum	*ERO1A*
hsa04145	Phagosome	*ATP6V1C2*
hsa04150	mTOR signaling pathway	*ATP6V1C2*
hsa04640	Hematopoietic cell lineage	*CD24*
hsa04657	IL-17 signaling pathway	*S100A7*
hsa04721	Synaptic vesicle cycle	*ATP6V1C2*
hsa04910	Insulin signaling pathway	*PPP1R3C*
hsa04924	Renin secretion	*CLCA4*
hsa04931	Insulin resistance	*PPP1R3C*
hsa04962	Vasopressin-regulated water reabsorption	*AQP3*
hsa04966	Collecting duct acid secretion	*ATP6V1C2*
hsa04972	Pancreatic secretion	*CLCA4*
hsa05110	Vibrio cholerae infection	*ATP6V1C2*, *ERO1A*
hsa05120	Epithelial cell signaling in Helicobacter pylori infection	*ATP6V1C2*
hsa05146	Amoebiasis	*SERPINB3*
hsa05165	Human papillomavirus infection	*ATP6V1C2*
hsa05323	Rheumatoid arthritis	*ATP6V1C2*

KEGG, Kyoto Encyclopedia of Genes and Genomes. DEG, differentially expressed gene.
[a] DEGs identified in this study mapped to the KEGG pathway.
